# Persistent use of body mass index policies as a barrier to surgery: Prevalence and analysis of policies across England in 2025

**DOI:** 10.1177/13558196251405207

**Published:** 2025-12-01

**Authors:** Kevin Ofosu, Katie Whale, Joanna McLaughlin

**Affiliations:** 1 Population Health Sciences, Bristol Medical School, 1980 University of Bristol, Bristol, UK; 2 NIHR Bristol Biomedical Research Centre, 1980 University Hospitals Bristol and Weston NHS Foundation Trust and University of Bristol, Bristol, UK

**Keywords:** arthroplasty, obesity, policy inequalities

## Abstract

**Background:**

Integrated Care Boards (ICBs) in England are responsible for commissioning healthcare services and setting access policies for procedures such as hip and knee replacement surgery. While the National Institute for Health and Care Excellence (NICE) advises against body mass index (BMI)-based restrictions, many ICBs impose such criteria. This study examines the prevalence and content of these policies to understand their impact on equitable healthcare access.

**Methods:**

A qualitative content analysis was conducted to systematically evaluate the policies set by all 42 ICBs in England regarding access to hip and knee replacement surgery for patients living with obesity. Policies were collected from official ICB websites and Google searches, completed in February 2025, and categorised as: no policy, restrictive policy, and non-restrictive policy. The alignment of these policies with clinical guidance was assessed, focusing on their potential impact on equitable healthcare access.

**Results:**

Policy documents were identified for 41 ICBs: 26 included weight management guidance and 15 imposed BMI-based restrictions on joint replacement referral eligibility. Policies varied in naming, terminology, and specificity, risking inconsistencies in interpretation and implementation. Some ICBs (*n* = 3) had revoked BMI-based restrictions in recent policy updates, reflecting a shift towards individualised clinical assessments. These findings highlight variability in policy approaches and the evolving stance on BMI-related eligibility criteria.

**Conclusions:**

One third of ICBs still use policies that contradict NICE guidance by restricting access to joint replacement surgery for those with high BMI, while many others apply ambiguous language. These inconsistencies highlight concerns about policies that may influence equitable access to care. Further work is required to evaluate how BMI-related eligibility criteria are applied in practice and their impact on health inequalities.

## Background

Integrated Care Boards (ICBs) were introduced in England in 2022 to improve collaboration between National Health Service (NHS) healthcare providers and commissioners. At the time of writing there are 42 ICBs, each serving a population of between 0.5 and three million, and they have a statutory function to improve NHS outcomes, experience, and access, and to reduce health inequalities. ICBs have autonomy in setting local healthcare policies, including criteria for surgical access.^1^ This flexibility, however, has led to inconsistencies in eligibility criteria for procedures such as elective joint replacement surgery.^2^ One major concern is the continued use of body mass index (BMI) thresholds to determine eligibility, despite the National Institute for Health and Care Excellence (NICE) guidance advising against such restrictions.^3^ The most recent policy audit found that in 2021, 42% of Clinical Commissioning Groups (CCGs), the NHS bodies responsible for commissioning local healthcare services before the formation of ICBs,had a restrictive BMI-related policy for hip and knee joint replacement referral.^2^

Hip and knee replacements are amongst the most common elective surgical procedures in the UK with over 200,000 performed annually.^4^ These operations are a well-established intervention for patients suffering from severe joint pain and functional impairment due to conditions such as osteoarthritis.^3^ Joint replacement can substantially improve patients’ quality of life by alleviating pain and restoring mobility. However, some ICBs impose BMI-based restrictions, requiring patients to attempt or achieve weight loss before being considered for surgery. While ICBs cite surgical risk reduction and cost-effectiveness as the rationale for policy use, they have sparked significant ethical and clinical concerns.^5,6^

Obesity is a growing public health issue in England, with over 28% of adults classified as obese and 63% as overweight or obese.^7^ The rationale behind focusing on BMI policies in joint replacement surgery lies in the debate over whether such restrictions genuinely improve patient outcomes or instead serve as a barrier to necessary medical interventions.^8^ Although obesity is associated with poorer acute post-operative pain responses and increased likelihood of needing revision surgery within 10 years, BMI alone is not a reliable predictor of surgical success.^9^ The strongest predictors of poor outcomes following joint replacement are levels of pre-operative pain and disease activity, depression and anxiety, and socioeconomic status.^10^ NICE guidance explicitly advises against the use of BMI as a barrier to surgical referral, emphasising that treatment decisions should be based on individual clinical need.^3^ Delaying or denying surgery due to BMI criteria can lead to worsening joint damage, increased reliance on pain medication, reduced overall mobility, and economic inactivity.^8^

In addition, there is significant variation in obesity levels across ethnic and socioeconomic groups. UK government data identifies the highest percentage of obesity in black adults, and obesity rates of approximately 35% in the most deprived areas compared to 20% in the least deprived.^7^ Therefore, BMI-based restrictions disproportionately affect socioeconomically disadvantaged groups, exacerbating existing health disparities.

The tension between equity, efficiency, and professional autonomy in surgical access is not unique to England. Internationally, the use of BMI criteria to determine eligibility for joint replacement has been debated, with regional and insurer-level policies similarly introducing BMI thresholds or “fitness for surgery” requirements as part of cost-containment or risk-reduction strategies.^11^ However, guidance from, for example, the American Academy of Orthopaedic Surgeons and the Arthroplasty Society of Australia instead emphasises individualised risk assessment, optimisation, and shared decision-making rather than exclusion based on BMI,^12,13^ and reinforces the global relevance of evaluating how commissioning policies translate into practice.

Research conducted in England in 2021 (before CCGs transitioned to ICBs) found that BMI restrictions were widespread and contributed to reduced access to surgery, and worsening of health inequalities.^2,14^ However, the current policy landscape remains unclear, particularly after the full establishment of ICBs. This study therefore seeks to reassess the policy landscape post-ICB implementation, examining the prevalence and content of BMI-related eligibility criteria for joint replacement surgery. The analysis aims to describe policy variation across England and to consider the potential implications for equitable healthcare delivery, while recognising that this study does not examine enforcement or patient outcomes.

## Methods

### Study design

This study used a qualitative, document analysis approach to examine the policies of ICBs in England in February 2025 regarding access to joint replacement surgery for patients living with obesity.^15^ The study design involved two key components:

#### Search strategy

A systematic retrieval of policy documents.

#### Policy analysis and summarisation

Categorisation and synthesis of extracted policy content.

### Data sources

The primary data sources for this study were publicly available policy documents retrieved from official ICB websites and supplementary searches using Google. These documents included clinical commissioning policies, elective surgery guidance, and related healthcare policies.

### Data collection and analysis

A systematic approach was used for document retrieval, review, and categorisation. Descriptive content analysis was used to categorise each document according to obesity policy.^16,17^ To ensure reliability and consistency in data collection and interpretation, each policy document was assessed by at least two independent reviewers. Discrepancies were resolved through discussion, and in cases where consensus could not be reached, a third reviewer provided a final decision.

### Search strategy

Official websites of all 42 ICBs were systematically explored, focusing on sections such as “Policies,” “Publications,” and “Clinical Guidelines”. When relevant documents were not directly accessible, website search functions were utilised, and documents related to orthopaedic surgery, clinical commissioning policies, and elective surgery guidelines were identified and reviewed.

Where no policy document was retrieved from an ICB’s website, targeted Google searches were completed combining ICB names with terms such as “joint replacement surgery policy,” “BMI restrictions,” and “clinical commissioning guidelines.” The first few pages of search results were reviewed to capture documents from third-party websites or publicly accessible databases. The searches were completed in February 2025.

### Policy categorisation and summarisation

A structured framework was developed using an inductive approach to classify the content of policy documents based on the degree to which weight management and BMI criteria influenced access to joint replacement surgery. Policies were assigned to seven categories, presented in Table 1. To enhance clarity, these sub-categories were subsequently grouped into three high-level categories to facilitate overarching analysis; policy not found (0), non-restrictive policy (1 & 2), and restrictive policy (3).

**Table 1.** Policy categorisation criteria and frequency.

Category	Sub-category	Detail	*n* (%)	
			Total = 42	
No policy	0	No policy found	1 (2.4%)	1 (2.4%)
Non-restrictive policy	1	Policy has no mention of BMI/weight/obesity/lifestyle	1 (2.4%)	26 (61.9%)
	2a	Mentions advice on weight and/or offers optional support but does not require weight management engagement or delay	9 (21.4%)	
	2b	States that conservative treatment, including weight management/lifestyle modifications, should be attempted first but lacks details on enforcement or evidence requirements	3 (7.1%)	
	2c	Specifies that conservative treatment should be attempted for a defined period and/or must be evidenced, mandated, or deemed ‘adequate'	13 (31.0%)	
Restrictive policy	3a	Requires patients to have attempted weight loss or accepted referral for weight management support before referral for surgery	7 (16.7%)	15 (35.7%)
	3b	Imposes a BMI threshold for surgical referral or an individual case-by-case application process, including upper limits on delays (e.g., mandatory weight loss over a specified period)	8 (19.0%)	

### Data extraction and processing

A structured data extraction form was developed to capture policy information, including policy document source, date, and criteria content (e.g., BMI thresholds, weight loss requirements, mandatory engagement in weight management programmes). The extraction form was piloted using documents from two ICBs and refined to ensure comprehensive data capture. An iterative approach was employed, allowing emerging findings to inform subsequent extractions and categorisations.

## Results

### Policy retrieval

Policy documents were identified for 41 of the 42 ICBs. 30 (71.4%) were located through the ICB website search, while 11 (26.1%) were only discoverable through wider Google searches.

### Variation in policy document naming

During the search and retrieval process, significant variations were observed in the naming and labelling of policy documents across different ICBs. Some policies were explicitly titled as “Clinical Commissioning Policy – Joint Replacement Surgery,” while others were embedded within broader documents such as “Elective Surgery Guidelines” or “Musculoskeletal Pathway Policies.” Additionally, terminology inconsistencies were identified, such as referring to BMI restrictions under “Preoperative Optimisation” rather than “Obesity Criteria”.

### Policy content

**Figure 1.**
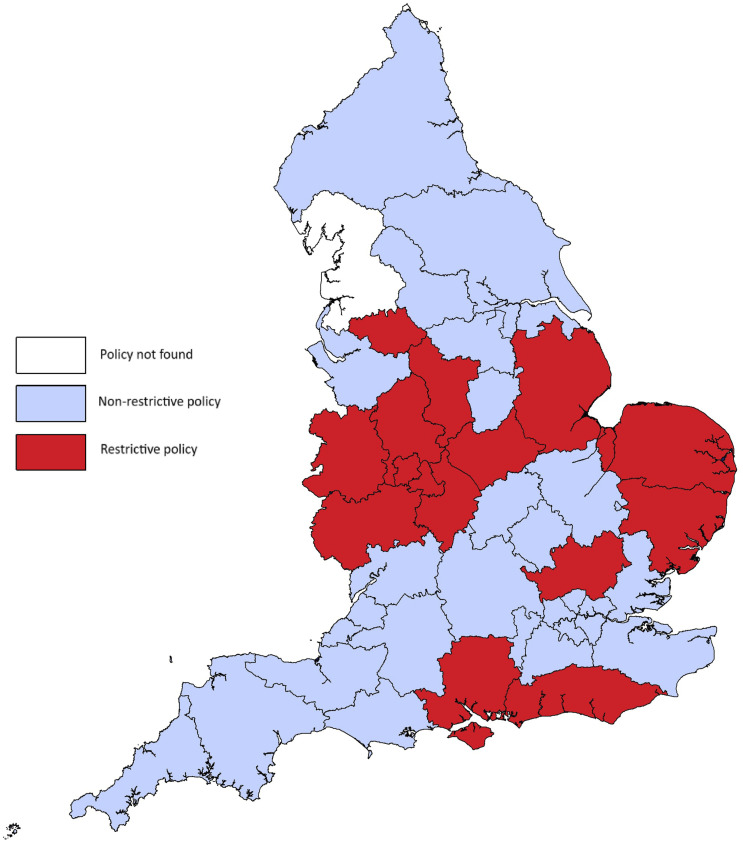
Geographical distribution of Integrated Care Board policies for hip and knee replacement surgery BMI eligibility by policy severity.

The frequency of ICB policy by category and subcategory is presented in Table 1, and the geographical distribution of the high-level ICB policy categories is displayed in Figure 1.

A third of ICBs (*n* = 15, 35.7%) implement restrictive criteria based on BMI or weight which limit access to joint replacement surgery. The remainder had non-restrictive policies which incorporated guidance on non-mandatory weight management as part of standard conservative treatment pathways (*n* = 25, 59.5%) or made no reference to BMI or weight (n = 1, 2.4%).

Detailed information on policy content and source for individual ICBs is provided in Tables S1 and S2 (Online Supplemental Material).

### Policy categories

#### No mention of BMI/weight/obesity/lifestyle factors

One policy (category 1, 2.4%) did not reference weight, BMI, or obesity as eligibility criteria for joint replacement surgery. This policy focused primarily on clinical necessity, without imposing additional conditions related to a patient’s weight status.

#### Mentions advice and optional support for weight loss

Nine policies (category 2a, 21.4%) provided general recommendations rather than strict criteria, advising patients to consider weight loss but without explicitly requiring evidence of weight management attempts or success. These policies often included vague wording, making it unclear whether weight loss was a firm requirement or merely encouraged. For instance, one policy stated, *“Patients should be encouraged to achieve a healthier weight before surgery,”* but did not clarify whether failing to do so would result in ineligibility. Another policy noted, *“Weight management support is available for patients who may benefit,”* yet did not specify whether participation in such programmes was mandatory or optional.

#### Conservative treatment including weight loss encouraged before surgery

Three policies (category 2b, 7.1%) recommended patients undergo conservative treatment, including weight loss efforts, before surgery could be considered. However, these policies lacked specificity in defining what constituted adequate weight loss or how long patients were expected to participate in conservative treatment. One policy stated, *“Patients should attempt lifestyle modifications before surgery,”* but did not outline clear benchmarks for success or failure. Other documents recommended *“a period of non-surgical management,”* but left the duration of this period unspecified.

Thirteen policies (category 2c, 31.0%) were more explicit in their requirements that conservative treatment must have been attempted for a defined period and/or must be evidenced, mandated, or deemed ‘adequate’. These policies did not include specific requirements on any engagement or success with weight management during this period, but indicated that conservative management “includes weight reduction where appropriate.”

#### Conservative treatment including weight loss required before surgery

Seven policies (category 3a, 16.7%) outlined strict weight loss requirements before joint replacement surgery, for example, one ICB requires that “*there has been evidence of weight reduction to an appropriate weight”*. Many of these policies lacked clear definitions in what amount of weight loss or engagement would suffice. For example, one policy required patients to *“demonstrate engagement in weight loss efforts before surgery may be approved,”* but did not provide measurable criteria for assessing engagement. Similarly, another policy stipulated that *“patients with a BMI above 35* kg/m^2^ *must attempt weight loss unless there are exceptional circumstances,”* without specifying what would qualify as an exceptional circumstance, nor what evidence of the attempt would be required.

#### Strict BMI thresholds for surgery

Eight policies (category 3b, 19.0%) set rigid BMI thresholds for surgery, often requiring a BMI below 35 kg/m^2^ or, in other cases, below 40 kg/m^2^ before a patient could be considered for joint replacement. Some of these thresholds were justified based on improving surgical outcomes, “*The morbidly obese (BMI >40* kg/m^2^*) and the super obese (BMI >50* kg/m^2^*) have complication profiles that may outweigh the functional benefits of total joint arthroplasty*”. Furthermore, some policies included ambiguous caveats that complicated their implementation. For instance, one policy stated, *“A BMI below 35* kg/m^2^ *is required for surgery except in cases where the risks of delaying surgery outweigh the benefits of weight loss,”* but did not specify how such risks and benefits should be assessed.

### Revoked policies

During the review, it was identified that some ICBs had previously imposed weight-related restrictions but had since revoked or amended these policies and made reference to these changes in their current policy documents. Notable examples included North East London ICB, where policy revisions aimed to *“reduce health inequalities”* by removing weight-based restrictions on joint replacement eligibility. Similarly, the Vale of York ICB lifted BMI restrictions concluding that they were “*unjustifiably restrictive*” following a *“robust clinical review”* that considered the appropriateness of such criteria in determining access to surgery.

Further changes were observed in Cheshire & Merseyside ICB, where an updated policy reflected a shift away from BMI-based thresholds, aligning with a broader movement to prioritise individual clinical assessments over rigid eligibility criteria. Published policy and communication documents detailing these revocation decisions are included in Table S2 (Online Supplemental Material).

## Discussion

### Summary of key findings

The analysis identified that a third of ICBs in England use restrictive policies which limit access to hip or knee joint replacement surgery based on BMI or weight loss. There was extensive variability in ICB policies with frequent lack of detail or use of ambiguous language in weight-related criteria. The findings suggest that while some ICBs maintain strict BMI thresholds for surgical referral, others are moving towards a more patient-centred and inclusive approach to surgical eligibility, but there are still changes needed to make all policies NICE-guidance compliant and reduce health inequalities.

### Interpretation in the context of existing literature

This study’s finding of significant variability in policy content aligns with previous research highlighting inconsistent approaches adopted by healthcare commissioners in England, raising concerns about equitable access to care.^18^ Studies in international settings have also evidenced variation in arthroplasty BMI-threshold use within countries,^19^ though the variation occurred at the level of individual surgeons than of commissioning bodies.^20,21^

Analyses in 2022 examined the impact of historic BMI policies on access to hip and knee replacement surgery across various regions in England.^14^ The researchers found that the introduction of BMI policies was associated with a decrease in surgery rates, affecting not only patients who exceeded the BMI thresholds but also those who technically met the criteria. This suggests that such policies may inadvertently reduce access to necessary surgeries for a broader population, potentially due to unclear policy wording or administrative barriers imposed on healthcare providers. The analyses also indicated that restrictive policies were linked to a decrease in surgery rates, particularly in groups facing socioeconomic deprivation, exacerbating health inequalities. In line with NICE guidance, rigid BMI cutoffs are therefore considered to act as a barrier to timely surgical intervention, prolonging disability and worsening quality of life for affected patients.

Recent analysis of demographics and clinical characteristics of those on elective surgical waiting lists in England confirm the high prevalence of obesity (29.4%) and that this is highest in the most deprived patient groups.^22^ BMI-related surgical access policies therefore affect many thousands of patients in England and are a concerning source of health inequalities.

### Implications for policy, practice, and future research

It must be acknowledged that there are additional considerations required for managing patients with higher BMI. They may require additional theatre time, specialist equipment, and trained staff, and surgical outcomes are influenced by both surgeon experience and hospital resources.^23,24^ However, policies should seek to support the equitable provision of care for patients in higher BMI groups, where shared decision-making has identified surgery as in the patient’s clinical interest, and avoid conflating economic or operational challenges with a direct rationale to restrict access in these groups.^25^ Enhancements in orthopaedic training to address surgical difficulty and increased operation length, and in-hospital bariatric equipment and facilities have been identified as pragmatic mitigations against the reluctance reported by some surgeons to manage patients with higher BMIs.^20^

The prevalence of restrictive policies, and the variability of policies regarding weight-related criteria for joint replacement surgery highlights the need for greater standardisation and transparency. The lack of uniformity creates disparities in patient access to care, with individuals in certain regions facing stricter eligibility criteria than others.

Healthcare providers play a critical role in interpreting and implementing ICB policies, yet the ambiguous language used in many policy documents creates challenges in clinical decision-making. For instance, some policies suggest that patients “should” be encouraged to lose weight before surgery but do not specify whether failing to do so would render them ineligible. Other policies require “engagement” in weight loss efforts but provide no clear criteria for what constitutes sufficient engagement.

This lack of clarity can lead to inconsistent application of policies, where some clinicians strictly enforce BMI cutoffs while others adopt a more flexible approach.

Orthopaedic surgeons retain professional autonomy, remaining the primary decision-maker in listing a patient for surgery, balancing policy guidance with clinical expertise, especially for patients presenting complex risks.^26^ Therefore, where eligibility criteria are less rigid, or there is no BMI policy in place, there may be informal, undocumented, Trust, department or even individual surgeon level criteria in use. For example, in a recent qualitative study, individual NHS surgeons described setting their own weight loss targets and conditions for patients on a case-by-case basis despite there being no formal ICB-level policy barrier to immediately listing the patient for surgery.^27^ In these cases, the right to a second opinion applies, and may protect some patients from unwarranted delays to their surgery, but this relies on patients’ agency and evidence shows that there are disparities in seeking a second opinion, such as in age, gender, health and socioeconomic status.^28^

Even where ICBs set a clear commissioning policy, the enforcement of such policies presents another variable in patient access. In the case of restrictive BMI threshold policies, it is assumed that clinicians cannot routinely supersede eligibility rules as these are contingent on remuneration for the payment attached to the treatment delivered. For example, one such ICB policy document notes “compliance with the criteria will be subject to regular clinical audits carried out or organised by the ICB. […] Where audit shows that the evidence is not available [that threshold criteria were met] it will not attract payment from the ICB.”^29^

Stigma is another important dimension, with restrictive policies shown to potentially reinforce weight bias in clinical encounters, further impacting patient care experiences. A USA study found that orthopaedic surgery is one of the clinical specialties in which high-weight bias is most prevalent, including explicit dislike of higher-weight patients.^30^ Research has shown that weight bias negatively influences clinical decision-making and patient experience,^31^ and there is concern that restrictive BMI policies reinforce weight stigma under the guise of clinical optimisation.^25^ Skilled and experienced communication is essential to ensure that clinician-patient discussions about the risks associated with high BMI and the benefits of pre-surgical weight management are constructive, respectful, and tailored to the orthopaedic context.^32^

Future research should examine how policies are enacted in clinical practice, whether hospital-level monitoring mechanisms exist, and how patients and surgeons experience the impact of BMI-based restrictions. In parallel, investigation of ICBs that have revoked restrictive criteria, such as those reported in this study, may provide insights into how such policy shifts affect actual service delivery, waiting times, and equity.

More broadly, BMI restrictions should be understood within debates about rationing and value-based commissioning, where research cautions that cost considerations may inadvertently exacerbate existing health inequities.^25,27^

### Strengths and limitations of the study

By employing a structured content analysis approach, this study systematically assessed and categorised ICB policies, providing detail of the current landscape regarding weight-related criteria for joint replacement surgery in England. The rigorous search strategy, multi-reviewer assessment process, and hierarchical categorisation ensured a replicable and transparent methodology for understanding policy variations across England.

Qualitative policy analysis allowed exploration of the inconsistencies in eligibility criteria, contributing valuable insights into disparities in healthcare access.

However, an important limitation is that this study focused on written policies rather than their enforcement; referral documents and specific implementation guidance were not included in the analysis. The extent to which policies are monitored or applied in clinical practice remains unclear, and informal practices in decision-making were outside the scope of this study. The perspectives of patients, clinicians, and commissioners were also not captured, yet these may provide critical insights into how policies affect experiences of care.

## Conclusion

This study provides a comprehensive analysis of the variability and ambiguity in ICB policies regarding weight-related eligibility criteria for hip and knee replacement surgery in England. The findings reveal high prevalence of restrictive policies which contradict NICE guidance, and a lack of uniformity across ICBs, with some regions enforcing strict BMI thresholds while others adopt more flexible or vague approaches. This inconsistency raises concerns about equitable access to surgery and the potential for health disparities, particularly for patients facing socioeconomic deprivation. Further research is required to examine how BMI-related eligibility criteria are enforced in practice, their impact on clinical decision-making, and the consequences for patients’ access to care.

## Supplemental Material

Supplemental Material - Persistent use of body mass index policies as a barrier to surgery: Prevalence and analysis of policies across England in 2025 Supplemental Material for Persistent use of body mass index policies as a barrier to surgery: Prevalence and analysis of policies across England in 2025 by Kevin Ofosu, Katie Whale, Joanna McLaughlin in Journal of Health Services Research & Policy

## Data Availability

All data are publicly available. Links to the policy documents are provided in Table S2 (Online Supplemental Material).*

## Appendix

### Abbreviations

BMI: body mass index
NICE: National Institute for Health and Care Excellence
NHS: National Health Service
ICB: Integrated Care Board
CCG: Clinical Commissioning Group
