# Supplementary material for: Persistent use of body mass index policies as a barrier to surgery: Prevalence and analysis of policies across England in 2025
Source: J Health Serv Res Policy. 2025 Dec 1;31(3):173–80. doi: 10.1177/13558196251405207 (PMC13263469; doi:10.1177/13558196251405207)
Supplement: Supplemental Material - Persistent use of body mass index policies as a barrier to surgery: Prevalence and analysis of policies across England in 2025 [file sj-pdf-1-hsr-10.1177_13558196251405207.pdf]

Journal of Health Services Research & Policy

Persistent use of body mass index policies as a barrier to surgery: prevalence and analysis of policies across England in 2025

Corresponding Author: McLaughlin, J.

Table S1 – Policy details by individual Integrated Care Board

INTERGRATED CARE BOARD	Policy Type	Policy document year	Relevant arthroplasty referral eligibility detail and extracts regarding BMI/obesity/weight from retrieved policies.
**EAST OF ENGLAND**			
**1.Bedfordshire, Luton & Milton Keynes**	2c	2023	Referral only when persistent pain not adequately relieved by at least 3 months of core treatments (weight loss if relevant). For patients who are overweight or obese and/ or active smokers: Primary care and community services should refer patients for weight loss and smoking cessation support at the earliest opportunity and in any case at the same time as referral to secondary care.
**2. Cambridgeshire & Peterborough**	2c	2023	Support offered to patients with BMI over 30, and over 35 referred to weight management service, e.g. healthy you. Evidence cited on page 3, refers to NICE guidance on revision risk.
**3. Hertfordshire & West Essex**	3a	2022	Referral should be considered when other pre-existing medical conditions have been optimised, and there has been evidence of weight reduction to an appropriate weight. The policy does also state later that NICE guidance says obesity should not be a barrier to referral.
**4. Mid & South Essex**	3a	2024	An extended course (at least 6 months) of non-surgical management to manage moderate to severe persistent pain has been exhausted and failed. This will include weight reduction and changing activity which NICE considers core treatments.
**5. Norfolk & Waveney**	2c	2024	If BMI>40 the patient should undergo 6 months conservative treatment, including a weight loss programme if BMI>40, though can proceed without this if GP documents that a worse outcome would result from the 6 months conservative treatment and risks are documented in the clinical letters.
**6. Suffolk & North East Essex**	3a	2021	They have a BMI ≤35kg/m2<br>OR<br>They have a BMI>35kg/m2 and have evidence of participating in a weight management programme in line with Policy 'Weight management and smoking cessation prior to elective surgery'. Patients whose BMI is >30 but ≤35kg/m2 should be advised that there is evidence that the outcomes of joint replacement surgery are better in people whose BMI is ≤30, and be offered support to lose weight.
**LONDON**			
**7. North Central London**	2c	2023	Primary care practitioners should ensure that the patient has meaningfully engaged with conservative management, where appropriate, prior to referral for hip replacement surgery'. Includes 'weight reduction, offering referral to appropriate services, where required'.

**8. North East London**	1	2019	North east London's (NEL) clinical reference group removed hip and knee surgery from previous BMI policy after considering feedback from a six week engagement exercise with patients, doctors and community groups.
**9. North West London**	2b	2023	Referral eligible when symptoms despite the use of non- surgical treatments such as adequate weight control treatments. Where appropriate, patients should be encouraged to reduce their BMI to <30 prior to surgery.
**10. South East London**	2b	2024	Access criteria include: 'The symptoms are refractory to non-surgical treatment (including analgesia, exercise, physiotherapy and **weight loss**, where appropriate'
**11. South West London**	2c	2023	Primary care should ensure that ALL the following conservative measures are attempted over a period of 6 months prior to referral for hip replacement surgery: Weight reduction where appropriate, particularly when the patient has a BMI greater than 35.
**MIDLANDS**			
**12. Birmingham & Solihull**	3a	undated but live	Patients with a BMI of 25 or more will be actively supported to engage with local weight management programmes to reduce their BMI. It is our consensus opinion that consideration should be given to delaying total joint arthroplasty in a patient with a BMI >40, especially when associated with other comorbid conditions, such as poorly controlled diabetes or malnutrition.' This means (for patients who DO NOT meet the specified criteria) the CCG will only fund the treatment if an Individual Funding Request (IFR) application proves exceptional clinical need and that is supported by the CCG.
**13. Black Country**	3b	2022	The ICB will fund THR, if the patient has a BMI of ≤ 35 and meets one or more of the following criteria(see page 6). If the patient has a BMI of over 35 and up to 40 and meets the criteria, the ICB will consider funding on a case-by-case basis.
**14. Coventry & Warwickshire**	3b	2022	The patient has a BMI below 35 supported by a primary care and/or community service referral. OR Patient has a BMI of 35 or over but mobility is so compromised that they are in immediate danger of losing their independence and that joint replacement would relieve this threat. OR Patient has a BMI of 35 or over but the destruction of their joint is of such severity that delaying surgical correction would increase technical difficulty of the procedure. If the patient does not meet any of the above criteria and has a BMI of 35 or over, they will be referred by their GP to weight management services and will be expected to engage with the services to achieve the required BMI. Should the patient's BMI fall below 35 then the patient would be eligible for surgery in line with the policy criteria. If this weight loss cannot be achieved the patient will be eligible for referral for surgery from two years after the documented date of the GP referral to weight management services for the purpose of weight loss prior to surgery.
**15. Derby & Derbyshire**	3a	2024	Criteria to be met before referral: Patient has engaged with referral to a lifestyle service for interventions to achieve weight loss if the person is overweight or obese.
**16. Herefordshire & Worcestershire**	3b	2023	Where a patient's BMI is above 35, the Commissioner will consider joint replacement surgery ONLY if there is evidence the patient has:<br>• Mobility so compromised that they are in immediate danger of losing their independence and that joint replacement would relieve this threat.<br>OR<br>• Joint destruction of such severity that delaying surgical correction would increase the technical difficulty of the procedure if delayed.

			OR<br>• Engaged actively with a weight management programme and achieved a 10% reduction in their weight.
**17. Leicester, Leicestershire & Rutland**	3b	2023	BMI <45. Referral should be made to the weight management Tier 3 service if BMI>35. Surgery is **not** offered for those with a BMI>45. For all : 3 months conservative treatment to include: discussion of weight management and smoking cessation as appropriate.
**18. Lincolnshire**	3b	2024	The patient has a BMI<35 and are a non-smoker. PATIENTS WITH A BMI >35: The referring clinician should stress the importance of trying to lose weight prior to surgery and support a referral to One You Lincolnshire. The patient should commit to a documented weight loss and exercise programme (this can be gentle exercise which will contribute to weight loss) programme for 3 months. Where weight loss is achieved and there is uncontrolled, intense persistent pain then the patient may be referred to an appropriate healthcare professional for consideration of surgery.
**19. Northamptonshire**	2c	2022	Patients should not be considered for surgery until it can be demonstrated that conservative methods have failed: Conservative management of hip pain due to osteoarthritis should include, where appropriate, weight reduction.
**20. Nottingham & Nottinghamshire**	2c	2024	No direct mention of weight/BMI/obesity/weight loss but does state that conservative treatments must have been tried for 3 months to include 'lifestyle modifications' where appropriate.
**21. Shropshire, Telford & Wrekin**	3b	2022	The patient has a documented BMI measurement of less than or equal to 40 at the time of referral or BMI over 40 including weight loss/management for 6 months. This is a temporary increase of the upper BMI limit to 40 for good reason due to covid, and will be reviewed and re-considered in the next policy update scheduled in 2023. For the duration of this change from <35 to <40 providers will be required to participate in a six monthly audit as part of the contract management process in order to ensure that there have been no adverse outcomes for this group of patients.
**22. Staffordshire & Stoke-on-Trent**	3b	2022	Patients with a BMI of 35 or more must be actively supported to engage with local weight management programmers to reduce their BMI, and only eligible for surgery if 'Has exhausted all appropriate non-surgical interventions'
**NORTH EAST & YORKSHIRE**			
**23. Humber & North Yorkshire**	2a	2024	Patients who have demonstrated good compliance to a comprehensive non-operative programme including NSAID's and analgesics, weight reduction, lifestyle modification and participation in therapy programmes. All patients who would benefit from a health improvement intervention to address weight management, smoking or other factors should be made a meaningful offer of support for this at appropriate stages in their conservative management and in all instances before referral is made for surgical assessment.<br>Patients with a BMI of >40 (the super-obese) are at increased risk of surgical complications and careful consideration should be given for surgery.
**24. North East & North Cumbria**	2c	2024	Hip replacement surgery will only be funded in accordance with the criteria specified below:<br>• The patient has accessed core (non-surgical) treatment options for at least 3 months as part of their management plan:

			includes:<br>o Access to facilitated interventions to achieve weight loss if the patient is overweight or obese.
**25. South Yorkshire**	2c	2023	Documentation required that 6 months of conservative measures have been tried, includes 'lifestyle adjustment' but no mention is made of weight/BMI/obesity.
**26. West Yorkshire**	2b	2020	Patients should engage with conservative management first, including weight reduction. Lifestyle factors, including weight, will be discussed as part of 'making every contact count' approach. Meaningful offer of support for weight management will be made.
**NORTH WEST**			
**27. Cheshire & Merseyside**	2a	2024	Advice and pre-operative guidance is given in relation to weight loss (if BMI >30 kg/m^2).
**28. Greater Manchester**	3a	2019	Supported activity and exercise, preferably a specific goals based supervised and evidence based physiotherapy programme for up to 3 months, and referral to a lifestyle service (or similar if available locally) for interventions to achieve weight loss if the patient is overweight or obese.
**29. Lancashire & South Cumbria**	0		
**SOUTH EAST**			
**30. Buckinghamshire, Oxfordshire & Berkshire West**	2a	2021	Requirement: Patients who have a BMI > 25kg/m2 are offered support and interventions to lose weight and those with BMI ≥ 35kg/m2 have been offered a recognised weight management programme. These should be documented. All overweight patients will be reviewed pre-operatively by the surgeon to ascertain medical fitness for surgery. Patient specific factors (including age, sex, smoking, obesity and co-morbidities) should not be barriers for surgery.
**31. Frimley**	2a	2024	Patients who have a BMI > 25kg/m2 are offered support and interventions to lose weight and those with BMI ≥ 35kg/m2 have been offered a recognised weight management programme. These should be documented.
**32. Hampshire & Isle of Wight**	3a	2024	If BMI is 35+ then evidence is required that referral has been made to a commissioned tier 2 or tier 3 obesity management programme.
**33. Kent & Medway**	2a	2024	To maximise the long-term functional benefit of joint replacement surgery and reduce the risk of complications during or following surgery, it is strongly advised to reduce BMI** to <30 prior to referral.<br>Patients with BMI ≥30 should be encouraged and supported to reduce their BMI both before and after surgery, including referral to weight management services where indicated.

**34. Surrey Heartlands**	2a	2022	Healthy lifestyle improvements: There is evidence to suggest that a healthy weight is associated with better outcomes pre/post-surgery. Evidence also shows a decline in the need for revision surgery when a patient weight is being kept within healthy parameters. It is anticipated that patients and clinicians in primary and secondary care will discuss options for weight loss where clinically appropriate including referral to weight management service – if available - prior to referral for surgery.
**35. Sussex**	3b	2024	Patients with a BMI greater than 35 kg/m2 should be routinely offered referral to a weight management service to reduce these risks. Patients who are morbidly obese (BMI > 40) should not normally be listed for hip joint replacement surgery unless all reasonable attempts have been made to reduce weight and there are compelling circumstances such as:<br>o Patients whose pain is so severe and/or mobility so compromised that they are in immediate danger of losing their independence and that joint replacement would relieve this threat<br>o Patients in whom the destruction of their joint is of such severity that delaying surgical correction would increase the technical difficulty of the procedure.
**SOUTH WEST**			
**36. Bath & North East Somerset, Swindon & Wiltshire**	2a	2022	Advice given on activity and exercise, diet, smoking cessation. Ensure conservative management has been attempted.
**37. Bristol, North Somerset & South Gloucestershire**	2c	2021	Fully engaged with conservative measures for a period of at least six months (clearly detailed throughout the patient's primary care record or via Musculoskeletal Services' clinic letters), as detailed within this policy (includes weight reduction where appropriate, particularly when the patient has a BMI greater than 35), and this has failed to improve the symptoms of the patient.
**38. Cornwall & Isles of Scilly**	2a	2017	Various 'In shape for surgery' policies. Patients with very high or very low body mass (BMI greater than 40 or lower than 18) are at additional risk in surgery, and this risk should be raised with them.
**39. Devon**	2a	2019	No body mass index (BMI) threshold will be placed on patients who require immediate referral for total knee and hip replacement surgery for clinical reasons. However, patients with a BMI of 35 or more will be actively supported to engage with local weight management programmes to reduce their BMI to maximise the functional benefit of surgery and reduce the risk of complications during or following surgery.
**40. Dorset**	2c	2019	Referral is only eligible if 'the patient has experienced persistent severe relevant pain despite adequate or maximally tolerated management in the primary and/or community setting' This should include 'Interventions to achieve weight loss if the person is overweight or obese'.
**41. Gloucestershire**	2c	2020	Symptoms persist despite the patient having fully engaged with conservative measures as defined by NICE Quality Standard QS87 (Quality Standard 7: Core treatments before referral for consideration of joint surgery) for a period of 3 months (unless the patient has severe persistent pain that is causing severe functional impairment which is compromising their mobility to such an extent that they are in immediate danger of losing their independence and joint replacement would relieve this, and conservative management as set out in this policy is contra-indicated).
**42. Somerset**	2c	2025	Offer advice on the following core treatments to all people with clinical osteoarthritis;<br>• Access to appropriate information

	• Activity and exercise<br>• Interventions to achieve weight loss if the person is overweight or obese<br>• Escape Pain http://www.escape-pain.org/<br>Patients with an elevated BMI of 30 or more are likely to receive fewer benefits from surgery and should be encouraged to lose weight prior to seeking surgery. In addition, the risks of surgery are significantly increased. Weight loss should be maximised prior to referral to Orthopaedic Assessment Services.

Corresponding Author: McLaughlin, J.

Table S2 – Policy document sources

INTERGRATED CARE BOARD	Link to policy document
**EAST OF ENGLAND**	
1.Bedfordshire, Luton & Milton Keynes	https://bedfordshirelutonandmiltonkeynes.icb.nhs.uk/~documents/policies/evidence-based-clinical-policies/hip-replacement-surgery
2. Cambridgeshire & Peterborough	https://www.cpics.org.uk/search?term=hip+replacement+policy&search=Search&searchType=all
3. Hertfordshire & West Essex	https://www.hweclinicalguidance.nhs.uk/clinical-policies/primary-hip-replacement/
4. Mid & South Essex	https://www.midandsouthessex.ics.nhs.uk/publications/srp-061-hip-joint-replacement/
5. Norfolk & Waveney	https://nwknowledgenow.nhs.uk/content/hip-arthroplasty-primary/
6. Suffolk & North East Essex	https://suffolkandnortheastessex.icb.nhs.uk/wp-content/uploads/2024/12/SNEE-ICB-CPP-Dec2024.pdf
**LONDON**	
7. North Central London	https://nclhealthandcare.org.uk/wp-content/uploads/2024/02/NCL-EBICS-Policy-v9.2-2.pdf
8. North East London	Revocation of restrictive policy:<br>https://www.healthwatchhackney.co.uk/news/hip-and-knees-dropped-from-surgery-access-plan/<br>https://democracy.havering.gov.uk/documents/s41716/Report%20-%20Aligining%20Commissioning%20Priorities.pdf
9. North West London	https://www.nwlondonicb.nhs.uk/application/files/3116/7664/3311/Hip_Replacement_v7.pdf
10. South East London	https://www.selondonics.org/wp-content/uploads/dlm_uploads/SEL-Treatment-Access-Policy-July-2024-v1.pdf
11. South West London	https://www.southwestlondon.icb.nhs.uk/wp-content/uploads/2022/07/NHS-South-West-London-evidence-based-interventions-policy-4.1.pdf
**MIDLANDS**	
12. Birmingham & Solihull	https://www.birminghamsolihull.icb.nhs.uk/application/files/6516/4872/1928/Policy_for_hip_replacement_surgery.pdf
13. Black Country	https://blackcountryics.org.uk/application/files/2816/8078/7236/BCICB_POLICY_-_Elective_Hip_Replacement_Surgery_v3_FINAL_Acc_Done.pdf

**14. Coventry & Warwickshire**	https://www.happyhealthylives.uk/clientfiles/files/document_library/Hip%20Replacement%20Policy.pdf
**15. Derby & Derbyshire**	https://www.derbyshiremedicinesmanagement.nhs.uk/assets/Clinical-Policies/Clinical_Policies/PLCV/ortho/Hip_and_Knee_Replacement.pdf
**16. Herefordshire & Worcestershire**	https://www.hwics.org.uk/application/files/2117/0904/4510/ICB_MSK_Surgery_and_Therapy_V1.5_Final_6.pdf
**17. Leicester, Leicestershire & Rutland**	https://leicesterleicestershireandrutland.icb.nhs.uk/llr-policy-for-hip-and-knee-replacement/
**18. Lincolnshire**	https://lincolnshire.icb.nhs.uk/documents/our-policies-and-procedures/clinical-governance/cg-003-prior-approval-policy/?layout=file
**19. Northamptonshire**	https://www.icnorthamptonshire.org.uk/download.cfm?doc=docm93jijm4n19770&ver=54137
**20. Nottingham & Nottinghamshire**	https://notts.icb.nhs.uk/wp-content/uploads/sites/2/2022/04/Value-Based-Commissioning-PolicyV1.4-2.pdf
**21. Shropshire, Telford & Wrekin**	https://www.shropshiretelfordandwrekinccg.nhs.uk/wp-content/uploads/Final-version-VBC-EBI-Policy-15.12.21.pdf
**22. Staffordshire & Stoke-on-Trent**	https://staffsstoke.icb.nhs.uk/your-nhs-integrated-care-board/our-publications/governance-handbook/all-policies/commissioning/icb-excluded-and-restricted-procedures-policy-v3-1/?layout=file
**NORTH EAST & YORKSHIRE**	
**23. Humber & North Yorkshire**	https://humberandnorthyorkshire.icb.nhs.uk/wp-content/uploads/2024/10/NY-CCG-Policies-New-Updated-18.10.24.pdf<br>**Revocation of previous restrictive policy:**<br>https://www.valeofyorkccg.nhs.uk/seecmsfile/?id=6719&inline=1&inline=1&inline=1&inline=1&inline=1&inline=1&inline=1&inline=1
**24. North East & North Cumbria**	https://northeastnorthcumbria.nhs.uk/media/dimlerzs/value-based-clinical-commissioning-policy.pdf
**25. South Yorkshire**	https://yourhealthrotherham.co.uk/wp-content/uploads/2023/05/FINAL_EBI_CFO_v.24_Final_27_March_2023-1.pdf
**26. West Yorkshire**	https://www.wypartnership.co.uk/application/files/3015/7925/4112/WYH_hip_replacement_policy_V1_12.19.pdf
**NORTH WEST**	
**27. Cheshire & Merseyside**	https://www.cheshireandmerseyside.nhs.uk/media/smrhlppb/pol-clin084-hipkneereplace-v1-final.pdf<br>**Revocation of previous restrictions:** https://www.hsj.co.uk/cheshire-and-merseyside-ics/ccgs-ration-hip-and-knee-surgery-for-obese-patients/7015887.article

**28. Greater Manchester**	http://northwestcsu.nhs.uk/BrickwallResource/GetResource/7eeddfcf-1297-4767-8b9f-2ae513eae601
**29. Lancashire & South Cumbria**	**None found**
**SOUTH EAST**	
**30. Buckinghamshire, Oxfordshire & Berkshire West**	www.bucksoxonberksw.icb.nhs.uk/media/4772/tvpc49-policy-update-primary-hip-and-knee-replacement-july-2021-v20.pdf
**31. Frimley**	https://fundingrequests.scwcsu.nhs.uk/wp-content/uploads/2024/04/BOBFPC-049-Primary-hip-and-knee-replacement-v2.0-2.pdf
**32. Hampshire & Isle of Wight**	https://fundingrequests.scwcsu.nhs.uk/hampshire-and-isle-of-wight-icb/policies-and-application-forms-hampshire-and-isle-of-wight/
**33. Kent & Medway**	https://www.kentandmedway.icb.nhs.uk/application/files/9817/1292/6708/KM_RaTC_April_2024.pdf
**34. Surrey Heartlands**	https://fundingrequests.scwcsu.nhs.uk/wp-content/uploads/2022/11/List-of-Procedures-with-Restrictions-and-Thresholds.pdf
**35. Sussex**	https://int.sussex.ics.nhs.uk/clinical_documents/sussex-cec-clinical-policy/
**SOUTH WEST**	
**36. Bath & North East Somerset, Swindon & Wiltshire**	https://bsw.icb.nhs.uk/document/hips-and-knees-policy/
**37. Bristol, North Somerset & South Gloucestershire**	https://bnssghealthiertogether.org.uk/wp-content/uploads/2019/07/Hip-Replacement-Surgery.pdf
**38. Cornwall & Isles of Scilly**	https://southwest.devonformularyguidance.nhs.uk/referral-guidance/key-messages/key-messages-archive/in-shape-for-surgery
**39. Devon**	https://onedevon.org.uk/download/hip-and-knee-replacement-surgery-in-obese-patients/
**40. Dorset**	https://nhsdorset.nhs.uk/wp-content/uploads/2022/11/Hip-replacement-EBI.pdf
**41. Gloucestershire**	https://www.nhsglos.nhs.uk/wp-content/uploads/2024/07/Elective-Hip-Replacement-Surgery.pdf
**42. Somerset**	https://nhssomerset.nhs.uk/wp-content/uploads/sites/2/Hip-Replacement-CBA-Policy-2425.V8.pdf
